# Effectiveness and cost-effectiveness of lifestyle interventions on physical activity and eating habits in persons with severe mental disorders: A systematic review

**DOI:** 10.1186/1479-5868-8-28

**Published:** 2011-04-11

**Authors:** Nick Verhaeghe, Jan De Maeseneer, Lea Maes, Cornelis Van Heeringen, Lieven Annemans

**Affiliations:** 1 Faculty of Medicine and Health Sciences, Ghent University, De Pintelaan 185 B-9000 Ghent, Belgium; 2 Faculty of Medicine and Pharmacy, Vrije Universiteit Brussel, Laarbeeklaan 103 B-1090 Brussel, Belgium

## Abstract

**Background:**

There is a high prevalence of overweight and obesity in persons with severe mental disorders and this has serious implications on the short and long term health outcomes of these patients. The aim of this review was to evaluate the effectiveness of lifestyle interventions targeting physical activity and eating habits in persons with severe mental disorders. Special attention was given if any of the included studies in the review also examined the cost-effectiveness of these health promotion interventions.

**Methods:**

A systematic search through the electronic databases Medline, Web of Science, CINAHL and Cohrane Library was conducted, and by hand-searching the reference lists of the retrieved articles from the electronic databases. Studies were included if they examined effectiveness and/or cost-effectiveness of lifestyle interventions targeting physical activity and eating habits in persons with severe mental disorders, with primary outcome changes in Body Mass Index and body weight.

**Results:**

Fourteen studies met the inclusion criteria. Weight loss and Body Mass Index decrease were observed in intervention groups in 11 studies. The difference in weight change between intervention and control groups was statistically significant in nine studies. Differences in mean Body Mass Index between intervention and control groups were statistically significant in eight studies. Five studies reported improvements in quality of life and general health. In none of the studies cost-effectiveness of lifestyle interventions was examined.

**Conclusion:**

Further research on both effectiveness and cost-effectiveness of lifestyle interventions targeting physical activity and eating habits in persons with severe mental disorders is required to assist in the development of new health promotion interventions in this population.

## Introduction

Overweight (Body Mass Index 25-29 kg/m^2^) and obesity (Body Mass Index >30 kg/m^2^) have become a serious global public health problem, and the prevalence of obesity is rapidly increasing [1]. Obesity is associated with lifestyle factors such as sedentary lifestyles and poor dietary choices [2,3] which contribute to major non communicable diseases, including cardiovascular disease (CVD), type 2 diabetes and certain types of cancer [4]. The burden of overweight and obesity is also increasing in low- and middle-income countries [5]. Where in the past obesity was mostly associated with the higher socioeconomic groups, now tends to shift towards the groups with lower socioeconomic status. In developing countries, the underprivileged people living in urban areas are especially affected by overweight and obesity [6].

Growing attention is given to the effects of 'healthy living interventions' targeting physical activity (PA) and eating habits. In the general population, the effectiveness of such interventions is already well established [7-9]. However, research on cost-effectiveness of 'healthy living interventions' does not provide clear evidence. The results of some studies suggest that specific lifestyle interventions for specific target groups are cost-effective [10-12]. Contrary, based on a systematic review, the authors concluded that there is currently no sufficient evidence to reliably compare cost-effectiveness results of individual studies and that the generalization of presented findings is restricted considerably [13].

In people with severe mental disorders (SMD) the prevalence of obesity is higher than in the general population [14,15]. Moreover, some studies provide evidence of differences in rates of overweight and obesity when gender is considered. In a study of the prevalence of overweight and obesity in bipolar patients, the results indicated that, in women with bipolar disorders, overweight and obesity were significantly more frequent, compared to reference women. In men, only rates of obesity were greater in patients than in reference subjects but rates of overweight were not [16]. In a study of 169 patients with schizophrenia or major mood disorder, 17.0% of women had Body Mass Index (BMI) in the obese III range (BMI >40 kg/m^2^), compared with only 4.0% in the general population. Among the male patients, 5.0% had BMI in the obese III range, compared with 2.0% in the general population [17].

In people with SMD, it is important to note that some atypical antipsychotics also have been associated with weight gain. The degree of weight gain can vary according to the type of antipsychotic medication [18].

Beside the higher prevalence of overweight and obesity, people with SMD also have more sedentary lifestyles, including less moderate to vigorous PA [19,20], and make poorer dietary choices, compared with the general population. Poor dietary choices include eating less frequently food recommended for large consumption (e.g. fresh fruit, vegetables, wholegrain bread, rice) and more instant meals [21-23] and fat [19] and having less variety of food in their diet [24,25].

Despite these negative conditions, patients suffering from SMD are less likely having their physical illnesses diagnosed and managed effectively. Barriers to effective physical healthcare include patient-related elements (poor treatment compliance, treatment refusal), the nature of the illness (social isolation and suspicion, physical symptoms unreported or masked because of high pain tolerance in some patients associated with the use of antipsychotics), physicians' attention principally focused on patients' psychiatric problems and physical complaints regarded as psychosomatic symptoms [26-29]. There is limited evidence describing that the presence of a MD does not affect the quality of physical health care. For example, no differences in quality of diabetes care were found between patients with schizophrenia or bipolar disorder and patients with diabetes without MD [30].

This raises even more concern given an increased risk of premature death in this population in comparison with the general population [26,31]. Premature mortality can be expressed by measuring the "years of potential life lost" (YPLL). The YPLL is based on the current mean survival age for a living cohort by age and by gender [32]. YPLL among persons with MD are described in several studies. In a study among 608 patients with MD, the mean YPLL for all causes was 32 years [33]. The results of a recent study identified a mean YPLL of 14.5 years in persons with MD compared with 10.3 years for the general population [34]. The risk of premature death can also be assessed by calculating the standardized mortality ratio (SMI). For example, in a retrospective cohort study, a SMI of 3.7 for patients with MD was found compared with the general population [35]. Respiratory disease, CVD and cancer are important causes of this premature mortality in addition to suicide [36-38]. As mentioned above, sedentary lifestyles and poor dietary choices are important risk factors for these non communicable diseases.

Therefore, people with SMD require careful baseline assessment and ongoing monitoring of physical health parameters [39]. The treatment and prevention strategies should include encouraging healthy lifestyles, smoking cessation, appropriate diets and levels of activity, with contribution of both mental health professionals and primary care providers [14,40,41]. The European Psychiatric Association stated that maintaining a healthy body weight and shape by healthy eating and regular PA is a key component in order to reduce the risk of some important somatic diseases such as CVD and to improve the overall health and well-being of patients [42].

The aim of this review was to evaluate the effects on weight, BMI and quality of life (QOL) of lifestyle interventions targeting PA and eating habits in persons with SMD. Special attention was given if any of the included studies in the review also examined the cost-effectiveness of these health promotion interventions.

## Methods

Initially, the following electronic databases were searched for the period 01-03-1990 until 01-03-2010: Medline, Web of Science, CINAHL and Cohrane Library using the search string '(Mental Disorders [MeSH] OR Severe Mental Illness OR Antipsychotic Agents [MeSH]) AND (Obesity [MeSH] OR Weight Gain [MeSH] OR Weight) AND (Lifestyle OR Intervention Studies [MeSH] OR Food Habits [MeSH] OR Physical Activity OR Fruit [MeSH] OR Vegetables [MeSH])'. The search was limited to randomized controlled trials, clinical trials, reviews and meta-analysis in English.

**Figure 1:**
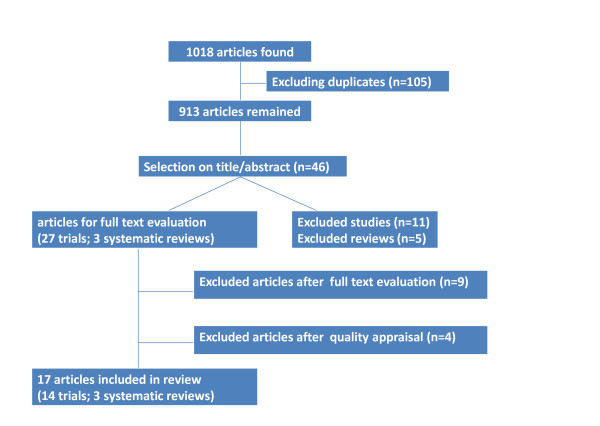
**Search strategy**.

One thousand and eighteen records were found (Figure 1). After excluding the duplicate records (n = 105) 913 references remained. One reviewer (the first author) assessed the relevance of the references. First, a selection was made on title and/or abstract. Studies were included if they examined effectiveness and/or cost-effectiveness of lifestyle interventions targeting PA and eating habits in persons with SMD. Studies were also included if participants were adults aged 18 and over with a DSM-IV diagnosis of schizophrenia, schizoaffective, depressive or bipolar disorder or 'severe mental disorder' in general. Furthermore, studies were included if the focus was on changes in weight and BMI through the application of psycho educational and/or behavioral interventions on PA and/or eating habits.

Studies were excluded if they examined the effect on weight and BMI of lifestyle interventions targeting PA and eating habits in general populations. Studies were also excluded if the primary outcome was not a mean change in weight and BMI by the end of the intervention. Studies examining the effect of a pharmacological intervention on body weight and BMI without lifestyle intervention were also excluded.

The selection based on title and/or abstract resulted in 34 references. Twelve additional references were found by hand-searching the reference lists of the retrieved articles from the electronic databases. Next, all non-systematic reviews (n = 5) were excluded, whereby 38 primary studies and three systematic reviews remained. As we were only interested in randomized and non-randomized controlled trials, all non-comparative trials (n = 11) were excluded. Full text of the remaining 27 primary studies was then evaluated in detail on the above mentioned in- and exclusion criteria. Nine trials were excluded. In two trials, participants were aged 15-44 years [43] and 16-50 years [44]. In three studies [45-47] changes in body weight and BMI were not considered as an outcome. In another study [48], changes in body weight and BMI were secondary outcomes. Two trials [49,50] reported no detailed data on changes in body weight or BMI. Finally, in one trial [51] changes in weight loss between persons with SMD and persons without psychiatric problems were compared.

After this selection, 18 primary studies remained for evaluation on quality. Study quality was assessed using a checklist developed by the Cochrane Centre (Dutch version) [52] and a checklist for the assessment of the methodological quality of both randomized and non-randomized studies of health care interventions [53].

Finally, 14 primary studies and three systematic reviews were appropriate for this review.

## Results

Three previous systematic reviews were identified [54-56]. The review by Faulkner et al. [54] systematically reviewed the literature of the effectiveness of interventions designed to control weight gain in schizophrenia. Sixteen studies met the inclusion criteria. Eight studies consisted of pharmacological interventions, which was an exclusion criterion for our review. The eight remaining trials consisted of behavioral/dietary interventions. Of these, two studies were also found by our electronic search. One [49] was excluded (no data on changes in BMI) and one [57] was included in our review. Six references were not found by our search. Four of these were conducted in the period before 1990 and consisted of dietary restriction treatment. Another study of the period after 1990 also consisted of a dietary intervention without behavioral intervention and one reference consisted of a letter to the editor.

In another systematic review of Faulkner et al. [55] 23 randomized controlled trials were included. Eighteen consisted of a pharmacological intervention, while the five remaining studies consisted of a cognitive/behavioral intervention. These five studies were also found in our search.

The study by Lowe & Lubos [56] systematically searched major databases for references about the effectiveness of weight management interventions for people with SMD receiving treatment with atypical antipsychotics. All studies (n = 8) included in this review were also found in our search. Six were included in our review.

### Primary studies - Participants

The total number of participants in the selected studies included 669 individuals (321 males, 328 females, 20 not specified). Mean age of the participants was 39.7 years. One study [58] provided no information about the mean age of participants. The target group of eight studies consisted of patients with schizophrenia or schizoaffective disorder, in three trials of patients with schizophrenia, in one study a combination of patients with schizophrenia, schizoaffective disorder, bipolar disorder or depression and in one study the target group were patients with bipolar disorder, schizoaffective disorder or depression. One study provided no detailed information on DSM-IV diagnosis of participants. The study population was defined as 'patients with mood or psychotic disorder'. In all studies, patients were treated with atypical antipsychotics such as olanzapine, clozapine, rispiridone.

The total number of 669 participants consisted of 361 individuals in the intervention groups and 308 in the control groups. Mean drop out rates were 20.6% (range 0.0 - 47.6%) in the intervention groups and 11.5% (range 0.0 - 33.3%) in the control groups. In three studies [58-60], no drop outs were observed in the intervention groups.

Nine studies recruited from outpatient settings, three from inpatient settings and two from a combination of both. Eight studies were conducted in the USA, while the remaining were from Italy (n = 2), Australia (n = 1), China (n = 1), Taiwan (n = 1), and South Korea (n = 1).

### Primary studies - Design

Among the 14 included studies, 11 were randomized controlled trials, while three were non-randomized controlled trials (table 1). In eight trials, assessment of the outcomes was made at baseline and endpoint, while in six studies multi evaluation points were used. In eight studies, assessment of the outcomes was based only on participants who completed the intervention period (per protocol). In the other six studies, outcome assessment was based on data of all participants (intention to treat).

**Table 1:** Design of studies included in the review

author	Design	intervention	study period	intervention period	individually/group-based
Scocco et al. [62]	randomized controlled trial	intervention on providing information and assessment of dietary topics compared to a control group not receiving intervention	24 weeks	8 weeks	individually-based
Evans et al. [64]	randomized controlled trial	intervention on education and setting specific goals on healthy eating and lifestyle compared to a control group not receiving intervention	6 months	3 months	individually-based
Littrell et al. [59]	randomized controlled trial	intervention on providing information and prompting self-monitoring of behavior on nutrition and exercise compared to a control group not receiving intervention	6 months	16 weeks	combination
Vreeland et al. [68]	non-randomized controlled trial	intervention on providing information and teaching on nutrition and healthy living compared to a control group not receiving intervention	12 weeks	12 weeks	combination
Weber & Wyne [58]	randomized controlled trial	intervention providing information and prompting self-monitoring of behavior on nutrition and exercise compared to a control group not receiving intervention	16 weeks	16 weeks	group-based
McKibbin et al. [61]	randomized controlled trial	intervention on exercise and providing information and prompting self-monitoring of behavior on nutrition and exercise compared to a control group not receiving intervention	24 weeks	24 weeks	group-based
Wu et al.[65]	randomized controlled trial	intervention on exercise and on providing information and prompting self-monitoring of behavior compared to a control group not receiving intervention	12 weeks	12 weeks	combination

author	Design	intervention	study period	intervention period	individually/group-based

Mauri et al. [63]	randomized controlled trial	intervention on providing information and teaching on nutrition compared to a control group receiving intervention in second study phase	24 weeks	12 weeks	combination
Wu et al. [60]	randomized controlled trial	intervention on exercise and assessment of food and caloric intake compared to a control group not receiving intervention	6 months	6 months	combination
Ball et al. [57]	non-randomized controlled trial	intervention on exercise and on prompting self-monitoring of food choices behaviour compared to a control group not receiving intervention	10 weeks	10 weeks	combination
Skrinar et al. [66]	randomized controlled trial	intervention on exercise and providing information and prompting self-monitoring of behavior on nutrition and wellness compared to a control group not receiving intervention	12 weeks	12 weeks	group-based
Kwon et al. [67]	randomized controlled trial	intervention on nutritional education and prompting self-monitoring of dietary behavior and exercise compared to a control group not receiving intervention	12 weeks	12 weeks	individually-based
Menza et al. [69]	non-randomized controlled trial	intervention on exercise and prompting self-monitoring of behavior on nutrition and exercise compared to a control group not receiving intervention	12 months	12 months	combination
Brar et al. [83]	randomized controlled trial	intervention on teaching various behavioral techniques on nutrition and exercise for weight loss compared to a control group not receiving intervention	14 weeks	14 weeks	group-based

### Primary studies - Weighing process

An analysis was performed to what extent information about the weighing process (time of weighing, the use of a weighing scale, clothing) was reported in the studies included in the review. In eight studies, no details about issues of the weighing process were described. Details about the time of weighing were described in only four studies [59-62]. In all of these four studies, participants were weighed in the morning. In only one study, details on the scale used for the weighing was described [63]. Two other studies reported that "the same scale" (without any details) was used. Details on the person(s) who performed the measurement of weight and height was described in three studies [58,62,63]. Information about the clothing was reported in four studies: "without shoes" [59,63], "in light clothing" [61], "wearing underwear" [62].

### Primary studies - Interventions

We were interested in studies on psycho educational and/or behavioral interventions on PA and/or eating habits in persons with SMD. Seven studies consisted of a psycho educational and/or behavioral intervention on PA and/or dietary management. In the remaining seven trials, the focus was not only on these kinds of interventions, but also included supervised exercise (walking or jogging). Topics on dietary management included education and discussion on eating habits with emphasis on energy intake/energy expenditure, and healthy eating. Topics on PA management focused on assessment of PA levels and discussion on changing activity levels and adequate amounts of exercise. Intervention were group-based (n = 4), individual (n = 3) or a combination of both (n = 7).

The mean duration of the interventions was 20 ± 10.8 weeks (range 10-52 weeks). The study period was equal to the duration of the health promotion intervention in 12 studies. In one study [64] the intervention period was 12 weeks, while the study period lasted 24 weeks. In another study [59] the intervention and study period lasted respectively 16 and 24 weeks.

In two trials the control group also received the intervention in a second phase of the study (cross-over design). In one study [62], the control group received no intervention from baseline until week nine. Afterwards, a cross over was made, whereby the controls also received the intervention between week nine and week 24. The study by Mauri et al. [63] had a similar design, as the control group also received the intervention between week 12 and week 24.

### Primary studies - Results of the interventions

First, changes in body weight and BMI were analyzed in intervention and control groups separately (table 2 &3). In the intervention groups, weight loss was observed in 11 studies. This was statistically significant in five studies and not statistically significant in one study. For five trials, no information on the level of significance of weight loss was provided. Weight increase was observed in three studies. In two studies [59,62], the increase was not statistically significant. The study by Evans et al.[64] provided no information on the level of significance of weight increase in the intervention group.

**Table 2:** Mean weight and BMI change from baseline to endpoint in intervention groups

author	number	baseline weight (kg)	endpoint weight (kg)	**baseline BMI (kg/m**^**2**^**)**	**endpoint BMI (kg/m**^**2**^**)**	changes in weight (kg)	**changes in BMI (kg/m**^**2**^**)**
Scocco et al. [62]^1^	10	75,33	76,32	27,53		0,99 ^NS^	
Evans et al. [64]	29	82,80	84,80	28,80	29,50	2,00 ^NI^	0,70 ^NI^
Littrell et al. [59]	35	81,12	81,49	26,26	26,39	0,37 ^NS^	0,13 ^NS^
Vreeland et al. [68]	31	100,20	97,48	34,32	33,34	-2,72 ^NI^	-0,98 ^NI^
Weber & Wyne [58]	8	86,64	84,20	33,00	32,05	-2,44 ^NS^	-0,95 ^NS^
McKibbin et al. [61]	32	100,83	98,52	33,60	32,90	-2,31 ^NI^	-0,70 ^NI^
Wu et al. [65]	32	64,70	63,40	24,60	23,10	-1,30 **	-1,50 **
Mauri et al. [63]^2^	22	83,90	80,30	30,00	28,70	-3,60 **	-1,30 **
Wu et al. [60]	28	78,40	74,20	30,43	28,84	-4,20 *	-1,59 *
Ball et al. [57]	21	107,91	105,60	36,90	36,20	-2,31 ^NI^	-0,70 ^NI^
Skrinar et al. [66]	15	97,10	94,90	32,90	32,30	-2,20 ^NI^	-0,60 ^NI^
Kwon et al. [67]^3^	33			26,81	25,31	-3,94 ^NI^	-1,50 ^NI^
Menza et al. [69]	31	100,20	97,20	34,30	32,60	-3,00 **	-1,70 **
Brar et al. [83]^4^	34	101,30	99,30			-2,00 **	-0,90 **

^1 ^Scocco et al. (2006): controls received intervention from week 9-24; results presented are the results after 8 weeks

^2 ^Mauri et al. (2008): controls received intervention from week 12-24; results presented are the results after 12 weeks

^3 ^Kwon et al. (2006): no information on baseline and endpoint weight, only on changes in weight

^4 ^Brar et al. (2005): no information on baseline and endpoint BMI, only on changes in BMI

* significant difference in weight or BMI change from baseline to endpoint in intervention groups (p < .001)

** significant difference in weight or BMI change from baseline to endpoint in intervention groups (p < .05)

Abbreviations: kg = kilogramme; BMI = Body Mass Index; NS = not significant; NI = no information on significance

**Table 3:** Mean weight and BMI change from baseline to endpoint in control groups

author	number	baseline weight (kg)	endpoint weight (kg)	baseline BMI (kg/m^2^)	endpoint BMI (kg/m^2^)	changes in weight (kg)	changes in BMI (kg/m^2^)
Scocco et al. [62]^1^	10	80,36	82,32	27,05		2,96 **	
Evans et al. [64]	22	80,20	86,20	27,20	29,20	6,00 ^NI^	2,00 ^NI^
Littrell et al. [59]	35	81,93	85,18	27,17	28,18	3,25 *	1,01 *
Vreeland et al. [68]	15	93,62	96,52	33,40	34,60	2,90 ^NI^	1,20 ^NI^
Weber & Wyne [58]	9	91,08	90,49	33,00	32,76	-0,59 ^NS^	-0,24 ^NS^
McKibbin et al. [61]	32	96,21	99,29	32,90	33,90	3,08 ^NI^	1,00 ^NI^
Wu et al. [65]	32	64,60	67,20	24,50	25,40	2,60 **	0,90 **
Mauri et al. [63]^2^	27	86,20	86,40	31,10	31,10	0,20 ^NS^	0,00 ^NS^
Wu et al. [60]	28	77,80	78,80	30,27	30,62	1,00 ^NI^	0,35 ^NI^
Ball et al. [57]	11	87,41	87,18	29,00	28,90	-0,23 ^NI^	-0,10 ^NI^
Skrinar et al. [66]	15	90,90	92,10	31,80	32,30	1,20 ^NI^	0,50 ^NI^
Kwon et al. [67]^3^	15			27,99	27,40	-1,48 ^NI^	-0,59 ^NI^
Menza et al. [69]	20	87,45	90,63	32,20	34,80	3,18 ^NI^	2,60 ^NI^
Brar et al. [83]^4^	37	98,90	97,80			-1,10 **	-0,50 **

^1 ^Scocco et al. (2006): controls received intervention from week 9-24; results presented are the results after 8 weeks

^2 ^Mauri et al. (2008): controls received intervention from week 12-24; results presented are the results after 12 weeks

^3 ^Kwon et al. (2006): no information on baseline and endpoint weight, only on changes in weight

^4 ^Brar et al. (2005): no information on baseline and endpoint BMI, only on changes in BMI

* significant difference in weight or BMI change from baseline to endpoint in intervention groups (p < .001)

** significant difference in weight or BMI change from baseline to endpoint in intervention groups (p < .05)

Abbreviations: kg = kilogramme; BMI = Body Mass Index; NS = not significant; NI = no information on significance

In the control groups, a decrease in mean body weight was reported in only four studies. This decrease was statistically significant in one study, not significant in another and two trials provided no information on significance of weight decline. In the other ten studies, mean endpoint weight in controls increased (in three a significant change, in one no significant change, and six reported no information on significance).

In the intervention groups, a decrease in the mean BMI was observed in 11 studies. The decrease was statistically significant in five of these trials. In one study the decrease was not significant, while for five trials no information on the level of significance was provided. Endpoint BMI was increased in two studies. In one trial, this increase was not statistically significant, while the other study reported no information on significance of BMI increase. One study [62] provided no information on mean endpoint BMI or changes in BMI neither in intervention nor in control groups.

In the control groups, the mean BMI decreased in only four studies (significant in one study, not significant in another study and no information in the other two). Mean BMI increased in nine trials. This increase was significant in two studies, and not significant in one study. No information on the level of significance was available for the remaining six studies. Only one study [65] reported confidence intervals around the changes in weight or BMI.

Secondly, differences in changes in body weight and BMI between the intervention and control groups were analyzed (table 4). The difference in weight change between intervention and control groups was statistically significant in nine studies and not statistically significant in five studies. Weighted average weight change based on sample size in the intervention groups was -1.96 ± 1.84 kg (-1.74%) versus +1.77 ± 2.12 kg (+2.28%) in the control groups. Differences in mean endpoint BMI between intervention and control groups were statistically significant in eight studies and not significant in three studies. In three trials, no information on significant changes in BMI was reported. Weighted average BMI change based on sample size in the intervention groups was -0.87 ± 0.69 kg/m^2 ^versus +0.64 ± 0.96 kg/m^2 ^in the control groups.

**Table 4:** Changes in weight and BMI between intervention and control groups

author	changes in weight (kg)	**changes in BMI (kg/m**^**2**^**)**
Scocco et al. [62]	-1,97 ^NS^	NI
Evans et al. [64]	-4,00 **	-1,30 **
Littrell et al. [59]	-2,88 **	-0,88 ^NI^
Vreeland et al. [68]	-5,62 **	-2,18 **
Weber & Wyne [58]	-1,85 ^NS^	-0,71 ^NS^
McKibbin et al. [61]	-5,39 *	-1,70 *
Wu et al. [65]	-3,90 *	-2,40 *
Mauri et al. [63]	-3,80 **	-1,30 **
Wu et al. [60]	-5,20 *	-1,94 *
Ball et al. [57]	-2,08 ^NS^	-0,60 ^NS^
Skrinar et al. [66]	-3,40 ^NS^	-1,10 ^NS^
Kwon et al. [67]	-2,46 **	-0,91 **
Menza et al. [69]	-6,18 **	-4,30 **
Brar et al. [83]	-0,90 ^NS^	-0,40 ^NI^

*significant difference in weight or BMI change from baseline to endpoint in intervention groups (p < .001)

** significant difference in weight or BMI change from baseline to endpoint in intervention groups (p < .05)

Abbreviations: kg = kilogramme; BMI = Body Mass Index; NS = not significant; NI = no information on significance

In four studies, the intervention period was followed by a follow up period during which the outcomes 'weight' and 'BMI' were also assessed. In the intervention group, a significant increase in weight (+3.16 kg, p < .03) during the follow up period of 12 weeks occurred in the study of Scocco et al. [62]. In the study of Evans et al. [64] the intervention group had not gained any further weight during the follow up period of three months. A similar stable mean body weight (follow up period of two months) was reported by Littrell et al.[59]. In the trial of Mauri et al. [63], weight loss occurred during the intervention period (-3.6 kg), with a further weight loss during the 16-week follow up period (-4.5 kg between baseline and endpoint).

Finally, differences in changes in body weight and BMI between intervention and control groups were studied in relation to the design of the intervention (individually, group-based or a combination of both) and to the duration of the interventions.

Seven studies consisted of a combination of an individual and a group-based intervention. In six of these studies significant changes in weight between intervention and control groups were found. Furthermore, two of three individual and one of four group-based interventions produced statistically significant weight changes between intervention and control groups. Similar results were found for changes in BMI between intervention and control groups. Changes in BMI were statistically significant in five of seven studies consisting of a combination of an individual and group intervention.

A significant difference in changes in body weight and BMI between intervention and control groups was found in five of six trials with an intervention period of 12 weeks. In the two trials with an intervention period of 24 weeks and one trial with an intervention period of 52 weeks, a significant difference in changes in body weight and BMI was found.

Among the eight studies in which assessment of the outcomes was based only on participants who completed the intervention period (per protocol), the differences in body weight and BMI between intervention and control groups were statistically significant in only four studies. In respectively five (body weight) and four (BMI) of six studies, in which outcome assessment was based on data of all participants (intention to treat), differences between intervention and control groups were statistically significant.

### Primary studies - Effects on quality of life

Effects on QOL of psycho educational and behavioral interventions targeting PA and eating habits were examined in five studies. Evans et al. [64] found a statistically significant difference between intervention and control group in subjective improvement of QOL (p = .047) and in overall health (p = .023) as measured by the Clinical Global Impressions (CGI). In the study by Mauri et al.[63] the results of the Clinical Global Impressions Severity (CGI-S) in the total population showed a significant improvement from baseline to endpoint (p < .05), while no significant changes were shown for the Clinical Global Impressions Improvement (CGI-I). No statistically significant differences on the CGI-S and CGI-I were observed between the intervention and control groups. Furthermore, scores on the Quality of Life Enjoyment and Satisfaction Questionnaire-Short Form (Q-LES-Q-SF) significantly improved only in the control group (p < .05).

The CGI-scale was also used in the study by Ball et al. [57]. No statistically significant differences in CGI were observed between baseline and endpoint of the intervention. In the study by Skrinar et al. [66] only subjective ratings of general health (p < .05) and empowerment (p < .01) were significantly more improved in the intervention group than in the control group. There was a statistically non-significant improvement in health and well-being from the Health Survey SF-36 and subscales from the Lehman Quality of Life Questionnaire.

Finally, scores on psychological well-being, social relationship, and environmental domains did not show a significant difference between the intervention and control group in the study by Kwon et al. [67]. There was only a trend toward statistical difference in the physical health score changes between the intervention and control group.

### Primary studies - Cost-effectiveness of interventions

The cost-effectiveness of psycho educational, behavioral and/or exercise interventions targeting PA and eating habits in persons with SMD was examined in none of the studies included in this review. Only in four studies, there was a reference to topics of cost-effectiveness. In their conclusion Vreeland et al. [68] and Menza et al. [69] stated that cost-effectiveness of these kinds of interventions must be examined in further studies. Evans et al.[64] concluded that further research is necessary to investigate the most cost-effective 'dose' of intervention required. Scocco et al. [62] emphasize the need for further research in order to develop an efficacious approach with an advantageous cost-benefit ratio.

## Discussion

The results of this review demonstrate that small improvements in body weight, BMI and QOL are possible through psycho educational and/or behavioral interventions targeting PA and/or eating habits in persons with SMD.

In the intervention groups, weight loss and a decrease of BMI is observed in 11 studies. In the control groups, a decrease in mean body weight and BMI is reported in only four studies. The difference in weight change between intervention and control groups is statistically significant in nine studies. Differences in mean endpoint BMI between intervention and control groups are statistically significant in eight studies.

Beside the significance of results it is also important to give attention to the clinical relevance of these results. According to the UK Department of Health [70] reductions in body weight of 5.0% or more are considered to greatly reduce the risks of physical health problems. In the included trials in our review, no study achieved this target. Moreover, only in a limited number of studies the weight loss resulted in changes of BMI classification (e.g. from obesity to overweight or from overweight to normal weight). In this sense, participants in the studies included in the review may lose weight following a lifestyle intervention but still remain in the overweight or obesity class. It can thus be questioned to what extent the BMI is useful to identify the risk for developing CVD. It appears that, according to the results of several studies, the measurement of the waist circumference and the waist-hip ratio is more appropriate than measuring the BMI to estimate the risk for future cardiovascular events [71,72]. It is important to note that in all 14 trials participants were taking atypical antipsychotics. It is well known that these drugs cause weight gain [18]. Furthermore, there is conclusive evidence that persons with SMD are more likely having sedentary lifestyles, making poor dietary choices and are more likely to smoke [21].

In eight studies included in the review, no information was provided about the weighing process (time of weighing, scale, clothing). The remaining six studies provided, however not always detailed, information about the weighing process. To minimize weighing errors, patients should be weighed at the same time of day using the same scale and in light clothing without shoes [73]. Including this information in the methods section of studies is important to enable an appropriate assessment of the results. According to the results of a study of weight measurement protocols no advantage was found about measurement of weight on two separate days compared with measurement on a single day [74]. However, as far as we know, literature on this topic is scarce and further studies evaluating weight measurement procedures are required.

There are some limitations to acknowledge. For this review, only references from the period 01/03/1990 until 01/03/2010 were included. In this way, it is possible that we miss relevant papers from the period before 1990.

The trials included in this review are frequently limited in terms of small sample sizes, short intervention periods and absence of long-term follow up. This raises questions about the generalization of the results to wider populations with mental disorders. In their review, Lowe & Lubos[56] concluded that the current literature on weight reduction interventions appears to provide limited evidence on the effectiveness either of psycho educational or of programs including educational and exercise components. They also concluded that more research is needed, with larger sample sizes as well as standardized outcome measures to determine and compare the effectiveness of these kinds of interventions.

The focus of the review considered 'healthy living interventions' in persons with SMD. This created a broad spectrum of 'healthy living interventions' topics and approaches. From a research perspective it may have been more appropriate only investigating one type of intervention. However, people with SMD share many risk factors, so it appears that general interventions will be more beneficial [75]. Weight management programs through healthy eating, exercise and tobacco cessation should be integrated into mental health care [76]. In this sense, it was decided to include randomized and non-randomized controlled trials with focus on health promotion interventions targeting PA and eating habits with primary outcomes changes in weight and BMI. Beside 'healthy living interventions' also pharmacological interventions to control weight gain in persons with MD have been evaluated. In a review of pharmacological and non-pharmacological interventions to control weight in persons with schizophrenia, the authors concluded that non-pharmacological interventions are preferable. The promising results in the non-pharmacological studies must however be tempered by weaker designs and small sample sizes used in these studies [54]. According to the results of a review on the mechanisms and management of antipsychotic weight gain in schizophrenia, it was concluded that pharmacological agents like orlistat and sibutramine have not been sufficiently evaluated in antipsychotic weight gain [77]

Despite the limitations, it is promising that small decreases of body weight and BMI in this population are possible. It appears that health promotion interventions targeting PA and eating habits in persons with SMD may be useful for prevention of weight gain. It is yet important to note that there may be some patients (outliers) that may derive tremendous benefit, but that the mean changes observed in the group are modest. Categorical outcomes would enable the calculation of the number needed to treat for the observation of a clinically significant benefit for the intervention. Only one study [65] reported confidence intervals around the changes in weight or BMI. Data on confidence intervals could however give insight in the ranges and thus in the solidness of weight changes.

Furthermore, persons with mental health problems usually want to learn more about healthy lifestyles and background theories of lifestyle interventions [78,79]. In a study of perceptions of barriers to and benefits of PA among patients with SMD, participants saw exercise as positive and desirable, with benefits for both physical and mental health [80]. This suggests that persons with SMD are prepared to participate in health promotion interventions.

Such findings support the integration of health promotion interventions targeting PA and eating habits into mental health care, whereby patients should be motivated to follow these kinds of interventions. When health promotion becomes a part of daily care, mental health professionals could play an important role in motivating their patients to participate. According to patients' perceptions mental health professionals can provide support, motivation, and structure and they feel comfortable with this support [81].

Elements of QOL were only investigated in five studies, providing no homogeneity of the effectiveness of 'healthy living interventions' on QOL and general health. Yet, improvements in QOL were, although not always statistically significant, observed. This is important because weight gain is associated with perceptions of poorer QOL and general health [82].

As far as known to the authors, this is the first systematic review of 'healthy living interventions' targeting PA and eating habits in persons with SMD, in which special attention was given if any of the included studies also examined the cost-effectiveness of these interventions. There is a growing need on health economic research in health care and health policy. Especially, attention is given to health economic evaluations of medicines and technologies. Recently, more attention is given to health economic evaluations of preventive health care. In general populations, research on cost-effectiveness of 'healthy living interventions' produces no conclusive evidence [12,13], which is likely explained by wide differences in program contents. In persons with SMD, no studies examining the cost-effectiveness of 'healthy living interventions' targeting PA and eating habits were found. Yet, such research has a great social value. Prevention has an economic cost, but it can also save money because diseases and complications can be avoided. Finally, prevention can produce healthy life expectation.

Eight of 14 studies included in this review were conducted in the USA. Because of differences in e.g. the management of health care, health insurance systems, and access to hospital care further studies, especially in European countries examining both effectiveness and cost-effectiveness of lifestyle interventions targeting PA and eating habits in persons with SMD are required to assist in the development of new health promotion interventions in this population. Concerning cost-effectiveness of interventions the viewpoint for the analysis (e.g. point of view of society, the Ministry of Health, the patient) should be carefully considered. Emphasis should also be put on long-term effects of these kinds of interventions.

## Conclusions

This review demonstrated that, however not always statistically significant, small improvements in body weight, BMI and QOL in persons with SMD are possible through health promotion interventions targeting PA and eating habits. In this sense, it appears to be relevant to integrate these kinds of interventions into the daily care of this population. Further research on both effectiveness and cost-effectiveness of lifestyle interventions on PA and eating habits in this population is required to assist in the development of new health promotion interventions. Additional qualitative research on perceptions of health promotion of both mental health professionals and patients appears to be relevant. This information can be useful when editing and implementing lifestyle interventions on PA and eating habits in mental health care.

## Competing interests

The authors declare that they have no competing interests.

## Authors' contributions

NV led the literature search strategy, retrieved, reviewed and assessed the studies on study quality and developed the paper. LA and LM participated in the design and coordination and helped to draft the manuscript, and revised it critically for intellectual content. KVH and JDM revised the manuscript critically for important intellectual content. All authors read and approved the final manuscript
